# Development of an Amorphous Selenium-Based Photodetector Driven by a Diamond Cold Cathode

**DOI:** 10.3390/s131013744

**Published:** 2013-10-11

**Authors:** Tomoaki Masuzawa, Ichitaro Saito, Takatoshi Yamada, Masanori Onishi, Hisato Yamaguchi, Yu Suzuki, Kousuke Oonuki, Nanako Kato, Shuichi Ogawa, Yuji Takakuwa, Angel T. T. Koh, Daniel H. C. Chua, Yusuke Mori, Tatsuo Shimosawa, Ken Okano

**Affiliations:** 1 Department of Material Science, International Christian University, 181-8585 Tokyo, Japan; E-Mails: g139004e@yamata.icu.ac.jp (T.M.); ichitaro@nt.icu.ac.jp (I.S.); g146025a@yamata.icu.ac.jp (M.O.); hisatoy@lanl.gov (H.Y.); ys3366399@gmail.com (Y.S.); konuki@mail.com (K.O.); nkato@astro.isas.jaxa.jp (N.K.); 2 Nanotube Research Center, National Institute of Advanced Industrial Science and Technology, 305-8565 Tsukuba, Japan; E-Mail: takatoshi-yamada@aist.go.jp; 3 Institute of Multidisciplinary Research for Advanced Materials, Tohoku University, 980-8577 Sendai, Japan; E-Mails: ogasyu@tagen.tohoku.ac.jp (S.O.); takakuwa@tagen.tohoku.ac.jp (Y.T.); 4 Department of Materials Science & Engineering, National University of Singapore, 117574 Singapore, Singapore; E-Mails: angel.koh@nus.edu.sg (A.T.T.K.); msechcd@nus.edu.sg (D.H.C.C.); 5 Department of Electrical Engineering, Graduate School of Engineering, Osaka University, 565-0871 Osaka, Japan; E-Mail: mori.yusuke@eei.eng.osaka-u.ac.jp; 6 Department of Clinical Laboratory, Faculty of Medicine, University of Tokyo, 113-8655 Tokyo, Japan; E-Mail: tshimo-tky@umin.ac.jp

**Keywords:** amorphous selenium, photodetector, carrier multiplication, diamond, cold cathode

## Abstract

Amorphous-selenium (a-Se) based photodetectors are promising candidates for imaging devices, due to their high spatial resolution and response speed, as well as extremely high sensitivity enhanced by an internal carrier multiplication. In addition, a-Se is reported to show sensitivity against wide variety of wavelengths, including visible, UV and X-ray, where a-Se based flat-panel X-ray detector was proposed. In order to develop an ultra high-sensitivity photodetector with a wide detectable wavelength range, a photodetector was fabricated using a-Se photoconductor and a nitrogen-doped diamond cold cathode. In the study, a prototype photodetector has been developed, and its response to visible and ultraviolet light are characterized.

## 1. Introduction

For centuries, scientists and engineers have attempted to replicate human vision and developed state-of-art devices such as complementary metal oxide semiconductor (CMOS) and charged couple devices (CCD). High-gain Avalanche Rushing amorphous Photoconductor (HARP) was one of the first successful photo imagers to exceed the sensitivity of a human eye [1], however, during its development, researchers found that a-Se possesses sensitivity beyond the visible light region; such as X-rays where a-Se X-ray imaging devices were proposed [2–4]. The unique carrier multiplication should lead to ultra high-sensitivity X-ray imagers, although, the unknown physics of carrier multiplication has prevented further progress; as of now, a lucky drift carrier multiplication mechanism has been proposed through empirical calculations and simulations [5,6].

In addition, detection of weak ultraviolet (UV) light has attracted much attention in various fields, including astronomic observation, flame sensors for security use, transparent photovoltaic devices, as well as medical imaging applications such as positron emission tomography [7–10]. A common UV detector is the avalanche photodiode made from silicon, however, this device has high thermal noise due to a relatively narrow band gap. While using wide-gap semiconductors can reduce the thermal noise, APDs made from wide-gap semiconductors are not sensitive to visible light, and are not suitable for spectrometric applications.

Amorphous selenium (a-Se) is a promising material for a high-sensitivity photodetector that has low thermal noise and a wide detectable wavelength range that covers visible to the UV [11], as well as X-rays [2–4]. The high sensitivity of a-Se based photodetector is explained by signal enhancement due to an internal carrier multiplication: this effect leads to quantum efficiency of greater than 100% [1,5,6], meaning that more than one carrier is generated per incident photon This study reviews the a-Se based photodetectors and their high-sensitivity operation utilizing carrier multiplication.

### 1.1. a-Se as a Photoconductive Material

Like other photoconductive materials, amorphous selenium generates electron hole pairs when it is exposed to light. This means that the current that flows through the material increases when it is illuminated. The increased current known as the photocurrent is caused by mobile carriers from the generated electrons and holes. However, under a fixed illuminance, the photocurrent is limited by trapping of the mobile carrier. The photocurrent depends on how far the free carriers travel before they are trapped in localized states. This is called the *range* and is defined as μτ, where μ is the drift mobility and τ is the lifetime, or the time before the excited electron is captured in a trap state. The μτ was measured by a method similar to the Haynes-Shockley experiment where transient current produced by a pulse light was measured in order to determine the electron and hole generation rate and transport efficiency. From these results, it was known that the *range* of holes were approximately in the order of 10^−6^ cm^2^/V with μ = 0.1∼0.2 cm^2^/Vsec and τ = 10–50 μs [12,13].

The *range* of electrons was one order of magnitude smaller than that of holes. Together with other amorphous semiconductors, the major challenge for the feasibility of a-Se devices was to overcome this recombination issue and acquire a longer *range*.

Alternatively, Kolomiets *et al.* introduced impurities such as S, Te, P, As and Tl and showed the sensitivity of a-Se [14]. Alloying a-Se with 0.5% arsenic causes hole transport to be trap-limited, however the electron transport was unaffected. Additional chlorine doping towards this composition in the ppm order, was found to enhance the hole transport [2,13]. Since As incorporation (0.2%–0.5%) is reported to prevent a-Se from crystallization [2,15], a-Se: 0.5%. As doped with Cl was a sufficient ratio for increasing both structural stability and hole transport property. This is usually referred to as *stabilized a-Se* and Table 1 compares the properties of undoped and *stabilized* a-Se photoconductor [13]. A more detailed analysis on the electronic properties of a-Se films, as well as comparison between other photoconductor materials, was reported in the literature [2,3,13].

**Figure 1.**
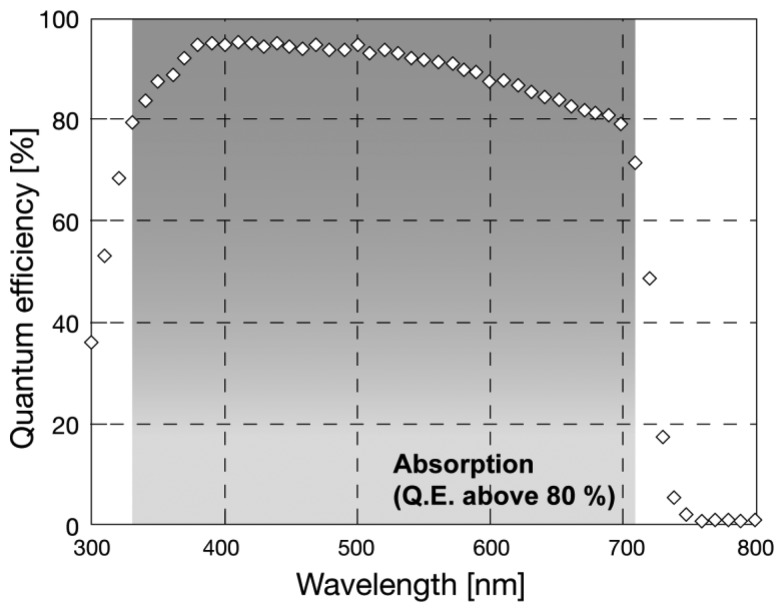
Spectrometric sensitivity chart of a-Se based photodetector after Kubota *et al.* [17] (labels translated into English). This photodetector used a CdSe layer to improve quantum efficiency at wavelength larger than 600 nm. The lower edge at around 350 nm should correspond to the absorption by glass substrate.

Sensitivity of a-Se was evaluated for different wavelengths by previous reports. The optical band gap of a-Se was reported to be 2.0 eV without tellurium (Te) incorporation [13], which was not suitable for detecting light with wavelength larger than 620 nm. The band gap can be narrowed down to 1.8 eV by Te incorporation, so that it could detect photons with wavelength of up to 689 nm [16]. The sensitivity to red light was further enhanced by later studies: Kubota *et al.*, introduced a CdSe layer to a-Se based photoconductor, which worked as photon capturing layer [17]. Figure 1 illustrates a sensitivity spectrum of a-Se based photodetector with CdSe layer after ref. [17].

**Figure 2.**
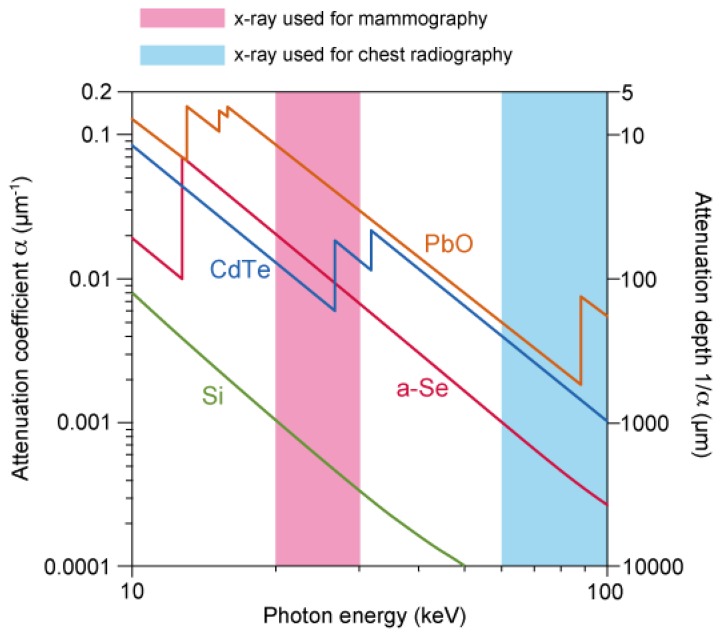
Attenuation coefficient of different X-ray photoconductors plotted in terms of photon energy [4].

The sensitivity to X-ray was studied in terms of attenuation coefficient, which is summarized by Kasap *et al.* in [4]. Although other photoconductors such as cadmium telluride (CdTe) and lead oxide (PbO) are superior by orders in high photon energy of > 30 keV, as shown in Figure 2, a-Se is widely used in the range 20–30 keV because of its high resistivity and ease of large-area deposition: since the detectability is determined by signal to noise (S/N) ratio, high-resistivity of a-Se can result in low dark current, giving high S/N ratio. The large area deposition using vacuum evaporation is another advantage, which can easily be applied to a flat-panel X-ray detector.

### 1.2. History of a-Se Based Photoconductive Devices

**Figure 3.**
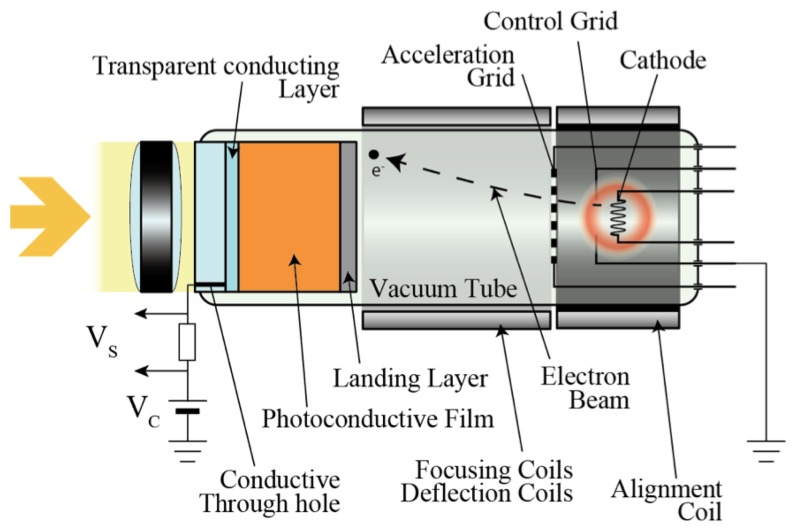
Schematic structure of a typical vidicon device [20].

The first photoconductive drum used for xerography consisted of an amorphous selenium thin film [18]. In the 1950s, various properties of a-Se, such as photoconductivity and photo generation, were reported [19]. As it can be imagined from the word “electrophotography (former term for xerography)”, the photoconductive properties were soon used for imaging devices and gave birth to vidicon tubes. As shown in Figure 3, a vidicon tube is a vacuum device that consists of a photoconductive target and a cathode array that enables it to scan and read out from the target.

**Figure 4.**
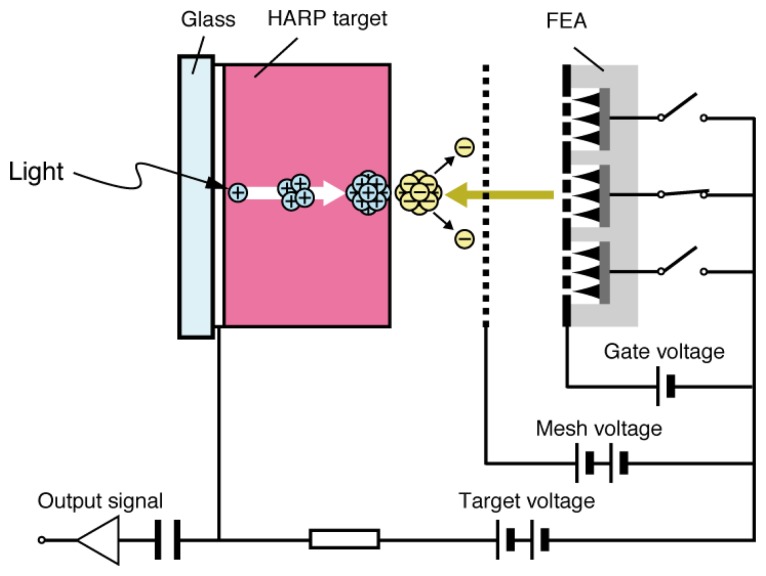
Schematic devise structure of FEA-HARP [21].

Se As Te target (SATICON), which was developed by Hitachi, was amongst the first vidicon targets that used amorphous selenium. Joined by the Japanese Broadcasting Corporation (NHK) research and development team, a vidicon tube called High-gain Avalanche Rushing amorphous Photoconductor (HARP) was invented in the 1980s utilizing impact ionization of the accelerated electrons and holes generated in the target film [1]. Although a-Se is not used for mainstream household devices, it has been utilized for high definition imaging technology. One of few drawbacks of the HARP camera tube was that it required a hot cathode for signal read-out, which led to high power consumption and limited lifetime, as well as restriction in downsizing due to the electron scanning mechanism [21–23]. A typical HARP camera tube was 10 cm in length, which prevents the device to be competitive to commercially available silicon-based imaging devices. Although much smaller versions of HARP camera tube have been developed by using field emitter arrays (FEAs), such as the one depicted in Figure 4, they still suffer from technological challenges such as limited resolution and dynamic range [21–23].

**Figure 5.**
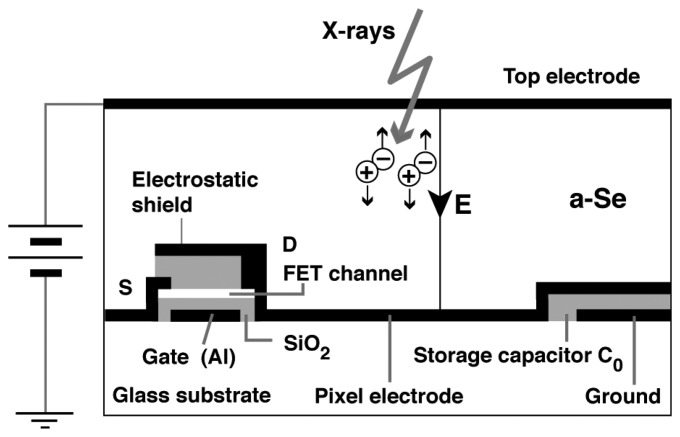
Schematic device structure of X-ray FPD by Kasap *et al.* [4].

An alternative application was suggested for a-Se. X-ray photoconductivity was discovered in the 1940s and opened up a realm of xeroradiography. After the success of the revolutionary Xerox 914 photocopier, a commercial xeroradiographic system named Xerox 125 medical imaging system has been developed. The development of electronic readout technology such as active matrix array was then combined with this xeroradiographic technique eliminating the necessity of toner development. Combined with thin film transistor technology, a new range of flat-panel X-ray detector was developed [2–4]. Figure 5 illustrates a structure of a-Se based flat-panel detector proposed by Kasap *et al* [4]. The a-Se based flat-panel detector (FPD) has found its application in medical diagnosis, however, its operation in high-sensitivity mode utilizing carrier multiplication has been limited due to uncontrolled breakdown in a-Se film and FET for signal read-out.

The cause of this breakdown is a local field enhancement in the device. Since the high-sensitivity mode requires electric field of 80–100 V/μm across the a-Se film [1,5,6], the operation voltage of a-Se FPDs are typically 1–1.5 kV. Such a high voltage often leads to breakdown of FET gate [24], as well as irreversible breakdown of a-Se layer due to a local field enhancement at the edge of metal electrode [25,26]. The local electric field can be reduced by introducing resistive interface layer, however, it sacrifices carrier transport property. Bubon *et al.* recently optimized the resistive interface layer, and reported a multiplication gain of 200 while maintaining the carrier transport properties [26]. In addition, fabrication of a hole-blocking layer prevents hole injection from back contact, which also help applying a high voltage across a-Se film [27].

Although these technologies seems promising for a-Se layers with limited thickness (∼a few tens of microns), better insulation techniques are needed for chest radiography and computed tomography: in such applications, X-ray of up to 100 keV is utilized, which is so permeable that it requires a-Se layer ten times thicker than typical HARP films (few hundred microns ∼ 1 mm) to maintain its sensitivity. In that case, vacuum-tube type devices have the obvious advantage of vacuum insulation.

Another remaining problem in a-Se based high-sensitivity X-ray detector is signal fluctuation due to the photo capturing process. Since the X-ray photons are much more energetic than those of visible light, the photons penetrate into a-Se photoconductor before interacting with it: since the penetration depth is limited by attenuation depth and is statistically distributed, this variation leads to signal fluctuation and may degrade image quality [28,29].

In order to achieve high-sensitivity a-Se based photodetector for wavelengths covering visible to X-ray, a novel device needs to be developed, which should solve the current problems in a-Se photodetectors. Toward that goal, a diamond cold cathode may be a promising choice to solve the current problems of FEA-HARP and a-Se FPD. With a negative electron affinity surface and high thermal conductivity, diamond emitters should supply sufficient emission current to provide a wide dynamic range. Furthermore, electron beams from diamond cold cathodes have small dispersion angles due to their geometrical flexibility, which can prevent a resolution loss at the emitter.

## 2. Development of a-Se Based Photodetectors

### 2.1. Nitrogen-Doped Diamond Cold Cathode [30–33]

**Figure 6.**
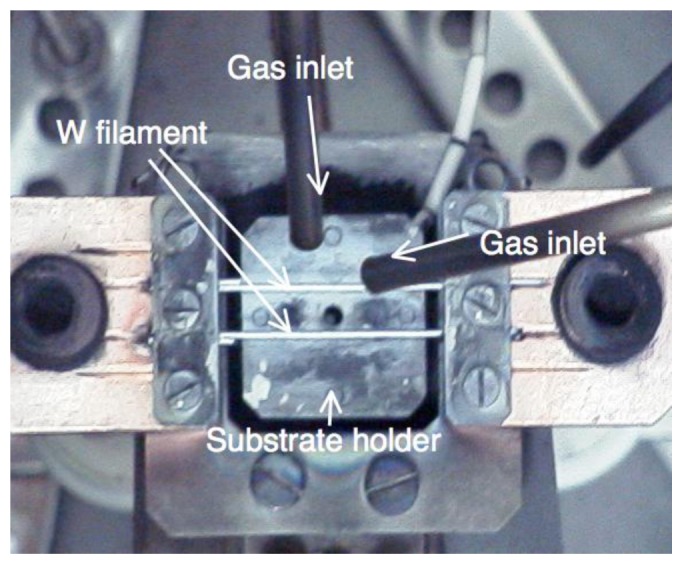
Hot filament chemical vapor deposition (HFCVD) system.

Nitrogen (N)-doped diamond films were grown using the hot filament chemical vapor deposition (HFCVD) technique under an acetone [(CH_3_)_2_CO] and hydrogen (H_2_) atmosphere [30]. A top view of the CVD apparatus is shown in Figure 6. A mixture of (CH_3_)_2_CO and H_2_ was introduced into a vacuum chamber while a rotary pump displaced the gas to balance the inside pressure. The chemical bonds of the gas molecules were broken by the thermal energy of the tungsten filament, and the carbon atoms were deposited on a substrate located just below the filament.

The deposition conditions were as follows: the filament temperature measured by an optical pyrometer was 2,300 °C and the substrate temperature measured by a thermocouple was 850 °C. The reactant gas ratio against H_2_ was 0.6 vol.% and the flow rate of the gas was 100 cc/min. The reaction pressure was held constant at 100 Torr during the growth. Urea [(NH_2_)_2_CO] was chosen for the dopant, because it has almost the same chemical structure as (CH_3_)_2_CO, the main source for the diamond growth and in addition, urea itself is not toxic. A saturated solution of (NH_2_)_2_CO and methanol (CH_3_OH) was diluted to l/10 with (CH_3_)_2_CO, and vaporized to be used as the reactant gas. The nitrogen concentration in the reactant gas can be estimated to be 30% assuming that all chemical compounds have the same vapor pressure. Films were grown for 2 h on 2.5 × 2.5 mm n-type (100) Si substrates, the surfaces of which were scratched with diamond paste with grain size of 1 μm in order to obtain high nucleation density for a continuous film.

The electron emission properties of the deposited diamond films were characterized by emission current (I) against the anode voltage (V) measurement [30]. The sample was mounted as the cathode in a vacuum system with a base pressure of ∼5 × 10^−7^ Torr. A glass plate coated with indium tin oxide (ITO) was placed 50 μm above the cathode as the anode using glass-fiber spacers. The leakage current in this measuring system was confirmed to be less than 2 × 10^−9^ A, which is the detection limit, from the fact that there was no current observed when the applied voltage was reversed, or when a molybdenum plate was mounted instead of diamond cathodes. The electric field (F) was calculated by dividing the anode voltage by the anode-cathode distance, and the current density (J) was obtained assuming that the emission occurred over the entire surface of the film. This assumption gives only the minimum current density; the emission is more likely to be occurring at particular sites rather than over the entire surface, and therefore a higher local current density must be expected. The surface morphology of the film observed by the scanning electron microscope indicated that the film surface was not flat and the size of each crystallite was 8 μm on average.

**Figure 7.**
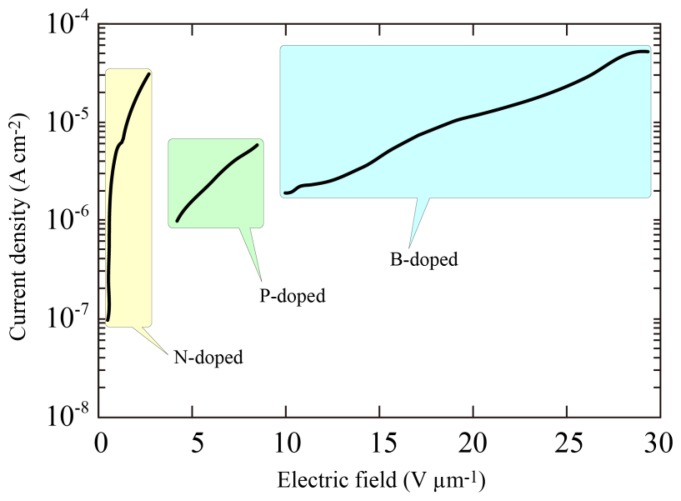
Comparison between the electron emission characteristics of nitrogen (N), phosphorus (P)-doped and boron (B)-doped diamond [30].

The remarkable J-F characteristics of the N-doped diamond indicated that the threshold field was less than 0.5 V/μm. Furthermore, no linear relation was observed in the J/F^2^ *versus* l/F characteristics suggesting that the current limit is not dominated by the standard tunneling theory proposed by Fowler and Nordheim. Comparison of the emission properties of N-, phosphorus (P)- and boron (B)-doped diamond films are shown in Figure 7. The surface morphologies of the P- and B-doped films were just the same as that of N-doped film, and there seemed to be no large variation in the enhancement factor evaluated from the radius of curvature. This led us to the view that the difference in the emission properties must be fundamentally due to the difference in impurities introduced into the diamond films. It was clear from the figure that the threshold field was much lower in the N-doped sample than P- or B-doped samples, indicating that incorporation of nitrogen in diamond played an important role in determining the emission characteristics.

**Figure 8.**
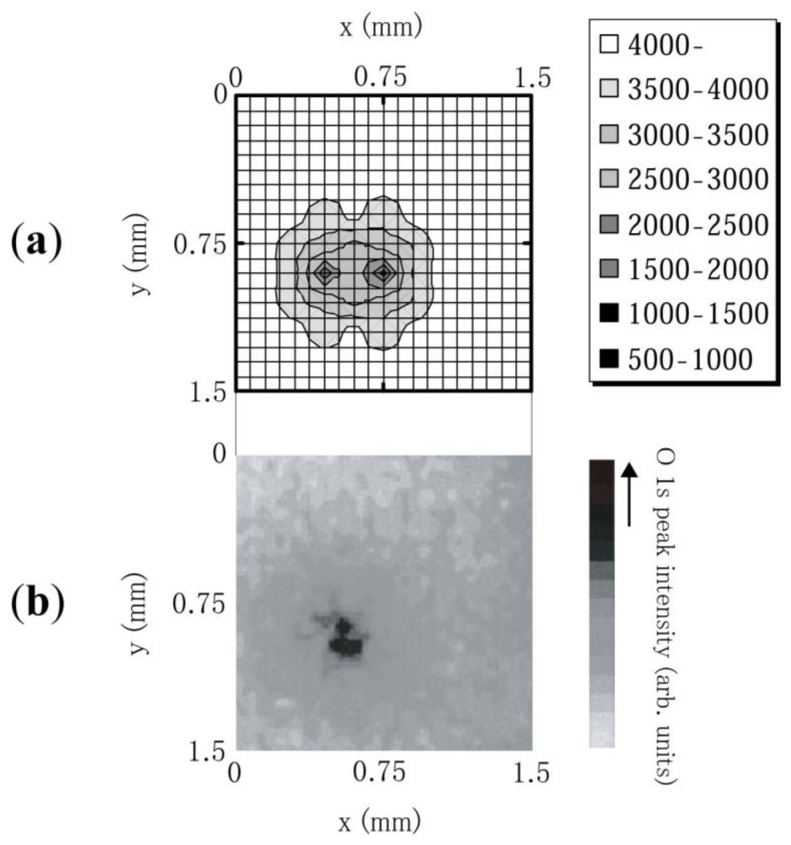
(**a**) Emission site map and (**b**) XPS intensity map (O1s) of N-doped diamond [31].

Using an electrochemically etched tungsten (W) needle placed 100 μm above the diamond surface as an anode, the emission site map (ESM) for polycrystalline N-doped diamond was observed in a vacuum system with a base pressure of 3.0 × 10^−8^ Torr [31]. The W needle can be moved in the x, y, and z directions and covers the whole sample size necessary to obtain ESM. X-ray photoelectron spectroscopy (XPS) was performed to determine the chemical state of surface carbon and any possible contaminants. MgKa; 1,253.6 eV operated at 150 W (15 kV, 10 mA), was used as the X-ray source in a vacuum chamber with a base pressure of 5 × 10^−10^ Torr (Shimadzu/Kratos AXIS ULTRA). In addition to the spectrum, the system enabled one to map the intensity for fixed energy with a spatial resolution of 15 um. The intensity map for polycrystalline diamond as shown in Figure 8b, indicated a strong correlation between the high O 1s intensity region and its emission site shown in Figure 8a. The result indicated the significant role of oxygen absorption on electron emission from N-doped CVD diamond [31].

**Figure 9.**
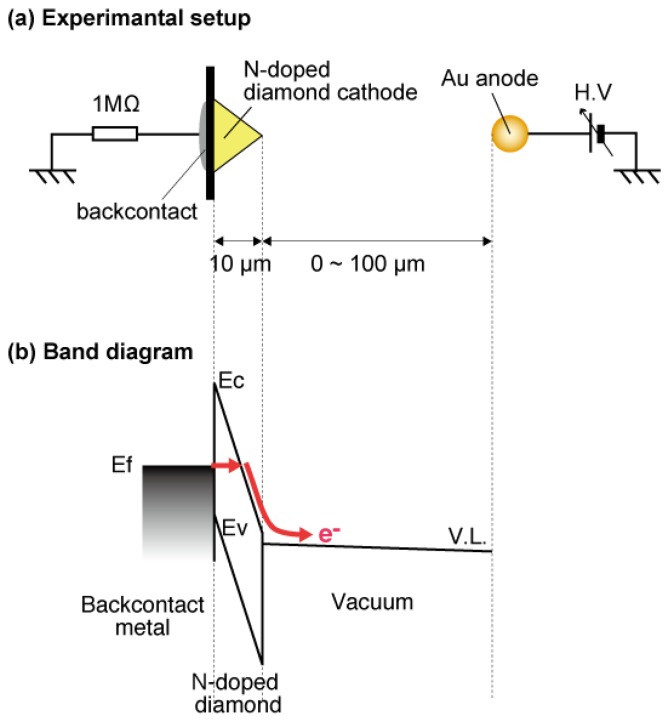
Schematic diagram of Metal-Insulator-Vacuum (MIV) type emission [32]: (**a**) experimental setup; (**b**) band diagram, where electron injection into diamond and electron emission from NEA are schematically drawn.

The electron emission mechanism of the high resistive N-doped diamond cathode was summarized as follows using metal-insulator-vacuum (MIV) model [32]. The fact that the field emission current was not limited by the diamond resistance even at relatively high current, suggested that the injection of carriers into the diamond increases exponentially with voltage. Therefore, a small current flow could lead to an exponential increase in the number of carriers available in the conduction band of the diamond through injection from the back contact. The negative/zero electron affinity of diamond allowed the exponential increase in electrons injected into the diamond from the back contact to be reflected in an exponential rise in the field emission current. Figure 9 illustrates the schematic diagram explaining MIV type electron emission. This process gave rise to the extremely low threshold field electron emission from the high resistivity N-doped CVD-grown diamond cathode. The results also implied that the N-doped CVD-grown diamond is one of the most appropriate materials for a low-threshold cold cathode operating in MIV-type electron emission mode.

**Figure 10.**
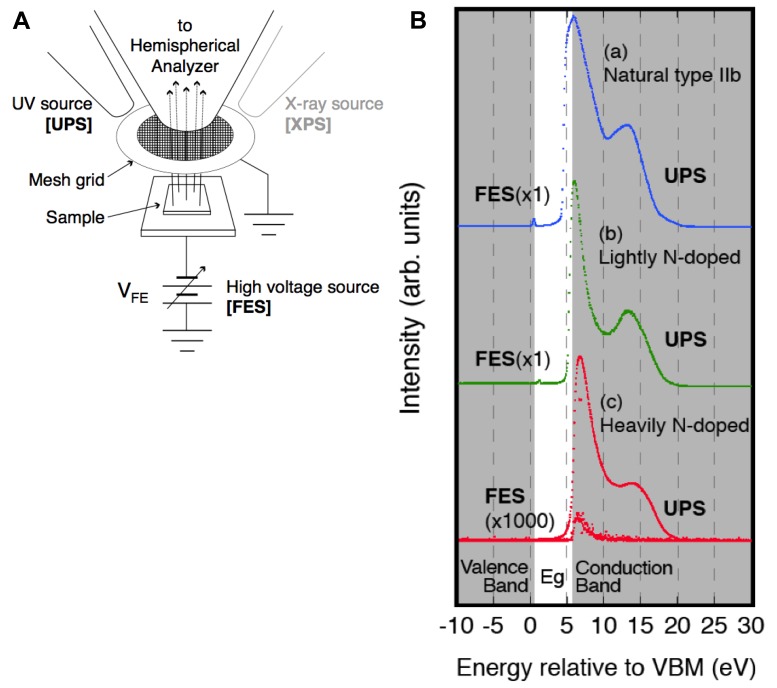
(**A**) Experimental setup and (**B**) UPS and FES combined spectra of (a) natural type IIb, (b) lightly N-doped and (c) heavily N-doped diamond, summarized after ref [33].

Combined XPS/UPS/FES measurements were performed on heavily N-doped polycrystalline CVD diamonds in order to clarify the mechanism of its extremely low-threshold electron emission [33]. The results clearly proved that the electrons are emitted from the conduction band (CB) of the diamond, utilizing NEA as shown in Figure 10.

The results could be well explained by the MIV-type electron emission, which the voltage applied between an anode and a cathode drops in diamond bulk, enhancing electron injection from the back contact metal to the CB of diamond due to quantum tunneling. Moreover, the results imply that this type of electron emission could be achieved using any material, provided that it possesses high resistivity and NEA surface. The low-threshold voltage, extremely narrow energy width of less than 0.3 eV, as well as the stable emission current suggests that this type of electron emission can be advantageous in a development of the next generation vacuum nano-electronic devices with long lifetime and high-energy resolution.

### 2.2. a-Se Based Photoconductive Film

#### 2.2.1. Deposition of a-Se Based Thin Films [10,20]

**Figure 11.**
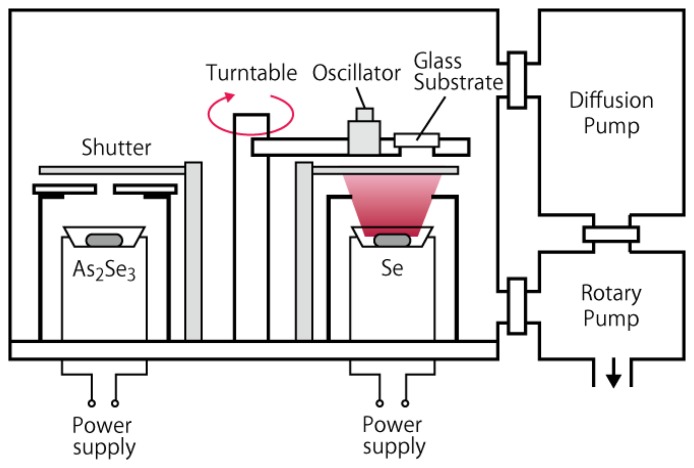
Schematic diagram of the rotational evaporation system used to deposit a-Se based films [10].

The a-Se based photoconductive films were deposited using vacuum evaporation, whose structure is schematically illustrated in Figure 11. The substrates were glass faceplates with electric through holes, on which indium-tin-oxide (ITO) transparent conductive films were deposited as back contact. The substrates were fixed on a turntable, which was equipped inside the evaporation system. Underneath the turntable were two molybdenum (Mo) boats that were filled with evaporation source. Two evaporation sources were used in the system: pure selenium (Se) pellet and powdered arsenic selenide (As_2_Se_3_), which were separately filled in each Mo boat and evaporated by Joule heat from the boat. The two Mo boats were placed on the opposite side of each other so that the substrate passed above the two sources alternately as the turntable rotated. The film deposited in this way should have multi-layer structure that consisted of Se and As rich Se layers. Incorporation of As was reported to increase the stability of amorphous structure, which could otherwise re-crystallize into trigonal selenium (t-Se) with smaller photoconductivity than amorphous counterparts [15,34]. The system could equip extra Mo boats for possible chlorine or tellurium doping, as reported in the literature [4].

The deposition was performed in the following manner: once the substrate and evaporation sources were loaded, the system was evacuated to 1.0 × 10^−3^ Torr using a rotary pump and then connected to a diffusion pump to be evacuated to 1.0 × 10^−6^ Torr. Prior to the deposition, the evaporation sources were preheated by increasing the electric current through the Mo boats. The current was increased linearly at 10 A/min, up to 40 A for Se boat and 45 A for As_2_Se_3_ boat, respectively. The maximum currents were kept for 5 min to ensure the sources to be vaporized, before the shutter above the boat was opened for film deposition. The deposition rate was monitored using a crystal oscillator, which was set alongside with the sample. The deposition was stopped when the oscillator indicated a 60 kHz drop, which corresponded to the film thickness of approximately 2 μm. During the deposition, the rotation speed of a turntable was kept at 80 rpm. After the deposition, the system was kept evacuated for another 1 h for cooling down. The a-Se based films were then taken from the chamber and annealed on a hotplate, with substrate side down. The temperature of the hotplate was increased from 45 to 65 °C by 5 °C steps, while the samples were annealed for 5 min at each step. The annealing temperature was optimized so that it helped a-Se structure shift into a quasi-stable state with stable current-voltage characteristics and resilience to thermal degradation [15]. Annealing with higher temperatures could cause the film to recrystallize into t-Se, which has lower resistivity and photoconductivity than a-Se.

#### 2.2.2. Film Composition and Stoichiometry Analysis [20,34]

X-ray photoelectron spectroscopy (XPS) measurements were carried out on a-Se and a-As_2_Se_3_ films in order to evaluate their composition and stoichiometry. A JEOL JPS-9010MX instrument equipped with a non-monochromated AlKa X-ray source was used for the measurement. The excitation power was 500 mW, and no slit for microanalysis was used so that the detection area is as wide as 10 mm in diameter, which almost covers the whole area of the sample. XPS spectra were recorded in the binding energy range from 0 to 400 eV at the base pressure of 5 × 10^−10^ Torr.

**Figure 12.**
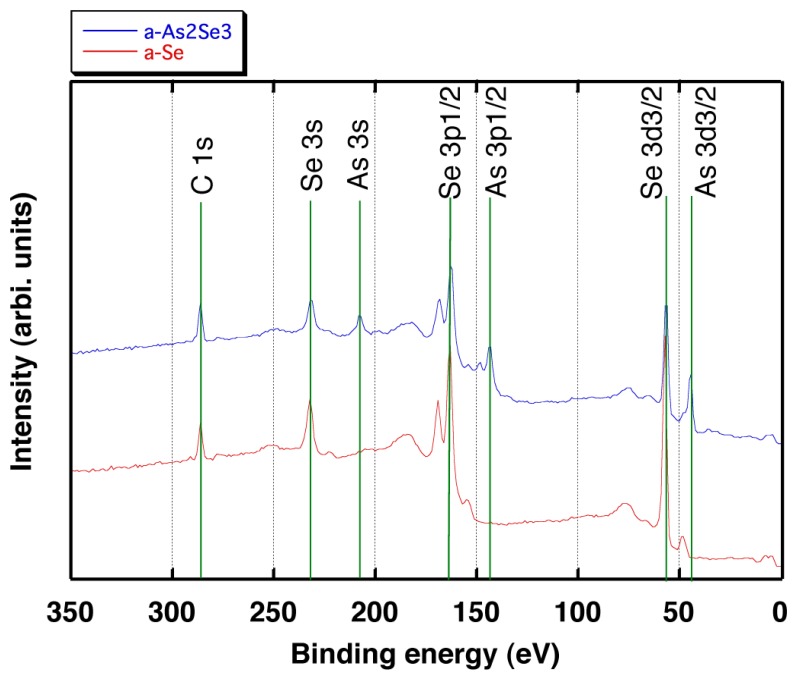
XPS spectra of a-As_2_Se_3_ (upper spectrum, blue) and a-Se (lower spectrum, red).

As a result, it is confirmed that the spectrum for the a-Se film contains peaks around 285 eV, 230 eV, 165 eV and 55 eV which can be assigned as C1s, Se3s, Se3p1/2 and Se3d3/2, respectively as shown in Figure 12. The spectrum for the a-As_2_Se_3_ film shown in Figure 12 exhibits 210 eV, 145 eV and 48 eV peaks which can be assigned as As3s, As3p1/2 and As3d3/2, respectively in addition to the peaks observed for the a-Se films [34]. The results indicates that using As_2_Se_3_ as the doping source gives rise to the addition of As into the deposited films.

Furthermore; the stoichiometry was evaluated by comparing the peak intensity ratio of the As3s to the Se3s peaks. First, the XPS spectrum was measured for As_2_Se_3_, which is used as source material and defined the obtained ratio (0.42) as the stoichiometry of 2:3. The peak intensity ratio was obtained as 0.45 for the deposited film and the stoichiometry was confirmed to be almost equivalent to the source material (2:3). The weak peak of As3s around 210 eV for the a-Se film should be the result of contamination since all the films were deposited in the same vacuum chamber. In addition, the stoichiometry of the a-As_2_Se_3_ film was also confirmed to be 2:3 by calculating the value using photoemission cross-sections reported in the literature [35]. The result proved that the films deposited using As_2_Se_3_ as a starting material contained almost the same amount of As compared to the source material, while those deposited using only Se contained negligible amount of As.

#### 2.2.3. Comparison of Photoconductivity between a-Se Films with Different Composition [15,20]

**Figure 13.**
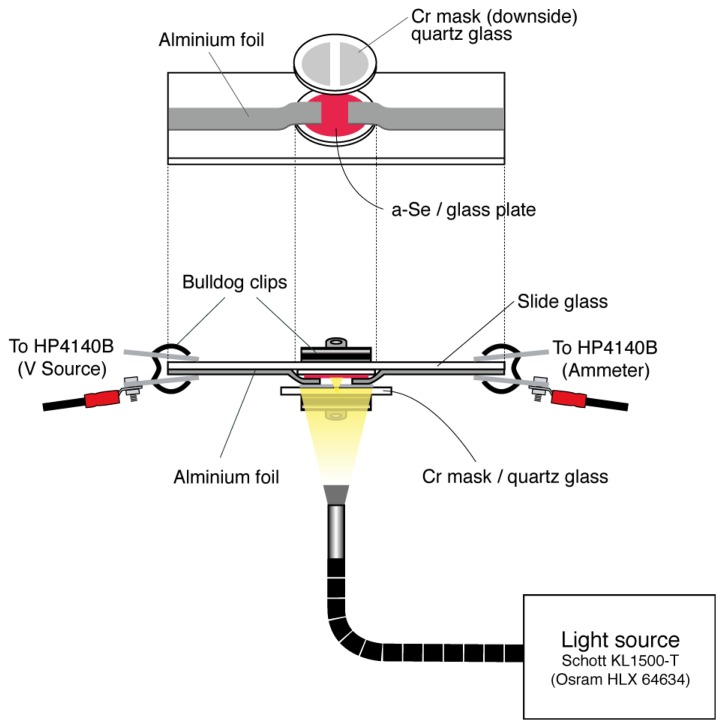
Schematic diagram of the setup used for I-V measurement [15].

Photoconductivity measurements were conducted using the deposited amorphous films [15]. Figure 13 shows the apparatus used in the measurement. As illustrated in the bottom view, the glass plate was placed on a slide glass and pieces of aluminum foil were pressed onto the surface of the evaporated selenium film. To reinforce the contact between the foil and the film, a quartz glass was pressed onto the top a clipped with a bulldog clip. The quartz glass had a layer of chromium sputtered onto its surface with a 1 mm gap in the middle to function as a mask in addition to ensure electrical contact. The end of the aluminum foil was clipped by another bulldog clip that had a crimping terminal that led to a HP 4140B, the voltage source and pico-ammeter. The significance of this module was that the photoconductivity measurement and the annealing can be done without removing the a-Se sample off the glass plate which drastically improves its reproducibility and also can be used in parallel with other measurements. The slide glass module was mounted with the film facing down as seen on the side view of Figure 13. The light source used for illuminating the sample was an OSRAM HLX 64634 mounted on a SCHOTT KL1500-T 150 W light source. The authors had first illuminated at the sample directly with the light, but the temperature of the sample surface increased rapidly and the fan of the light source caused noise. Therefore, a light guide was used to secure enough space between the measurement system and the light source. By placing the light source outside the shield box, which the measurement system was placed in, it could keep the temperature low, and cut off a substantial amount of noise. The distance between the tip of the light guide and the sample surface was kept at 43 mm in the setup, which provided the estimated illumination area of 38 mm in diameter sufficiently covering the whole sample.

**Figure 14.**
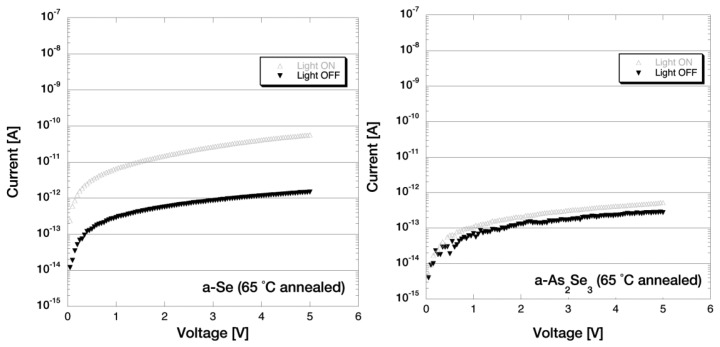
I-V characteristics of a-Se and a-As_2_Se_3_, after annealing at 65 °C [15].

**Figure 15.**
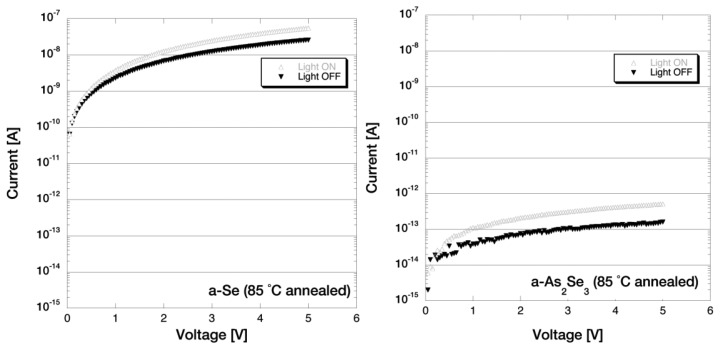
I-V characteristics of a-Se and a-As2Se3, after annealing at 85 °C [15].

The films were annealed on a Cu platform mounted on a hotplate under atmospheric pressure. A thermocouple was attached beside the sample to monitor the annealing temperature. The hotplate was preheated to ±1 degrees of the annealing temperature before the samples were mounted on the Cu platform. The samples used for this photoconductivity measurement was annealed at 60–85 °C in 5 °C steps for 5 min each. Note that the annealing conditions and the treatment of the samples were much the same to that of the optical microscope observation and the visible light spectroscopy measurements. Figure 14 shows the I-V characteristics for a-Se and a-As_2_Se_3_ films after annealing at 65 °C. The dark current of a-Se sample increased after each annealing step and it increased rapidly after 70 °C annealing. The dark current reached up to 10^−8^ A order after 85 °C annealing, which is shown in Figure 15.

The color of a-Se film changed from dark red to black in after a 65 °C anneal and also started to tarnish after a 70 °C anneal. After a 75 °C anneal, the surface changed into a gray, dull metallic color and the shape of the tip of the light guide became vague when looked through from the other side of the module. All the remained black areas turned completely gray after 80 °C and only a weak glow could be seen coming through the module.

Here we define the ratio between the dark and illuminated current (light/dark) as photoconductivity ratio. The ratio was 2.02 at 5 V for un-annealed film. The photoconductivity ratio increased to 38.12 after when it was annealed at 65 °C and decreased after higher temperature annealing. These results showed that a certain amount of annealing treatment could improve the photoconductivity of the a-Se film, but if annealed at temperatures higher than 65 °C, not only it resulted in the decrease of the photoconductivity ratio, but it also led to the degradation of the film. The previous Raman spectra results showed that the degradation corresponded to the crystalline t-Se state [34]. Compared to the previous visible spectrum and the optical microscopic results, it was clear that the photoconductivity ratio decreased as the crystallization progresses. Although the present result suggests a glass transition temperature at temperature between 60 and 70 °C, previous studies such as [36] reported a photo-induced structural change of a-Se film at temperature as low as 35 °C. These results suggest that Se may have several “metastable states”, where both photo-induced and heat-induced phase changes can occur. Further research in the phase transition property of a-Se should lead to optimum design of a-Se film with desired electronic property.

In contrast to pure a-Se film, I-V characteristics for As_2_Se_3_ films in Figures 14 and 15 showed much smaller changes throughout the annealing steps: despite the fact where the dark current of a-Se changed drastically, the dark current of a-As_2_Se_3_ was stable around late 10^−13^ A for any annealing temperature. Very carefully observing the color of the film, the already dark colored a-As_2_Se_3_ film could be seen to have darkened after each annealing. The same could be said towards the photoconductivity ratio where it did indicate a slight increase of up to 3.76 after 85 °C annealing, but was still much smaller than a-Se films.

From these results, great changes in the appearance and the photoconductive property could be observed for a-Se films and not for a-As_2_Se_3_ films, which was consistent with the result of optical microscope observation and visible light spectroscopy measurements.

It should be noted that with a certain amount of annealing treatment, where in this case around 65 °C, seemed to have improved the photoconductivity of the a-Se film. It could be explained as follows: the lattice vibration caused by annealing treatment led to the reassignment of the disordered selenium particle in the as-depo film. From the results of this experiment, the a-Se films fabricated under the conditions written above, could have reached a metastable state at a temperature of between 60 °C and 70 °C during when the color of the film was black and just before it started to tarnish, and also before the dark current increased rapidly. However the low energy potential around the a-Se metastable state could be easily overcome by applying more energy to the film, and thus a-Se shifted to a more lower energy state.

Two possible patterns could be conceived for the annealing-induced degradation of amorphous selenium. One possibility was that the degradation occurred from the interface of the film and the substrate glass, and gradually developed towards the surface of the film as more annealing treatment was applied. The other possibility was that the degradation occurred from the surface of the film, caused by some reaction between amorphous selenium and a substance in the atmosphere (e.g., oxygen, water *etc.*) and progressed into the depth of the film. The present results and our previous data showed a large decrease in the resistivity and a significant change in the optical microscope observation at higher annealing temperature, while the color of the film viewed from the top still remained a dark wine red, even after extra annealing at 90 °C and 95 °C for 5 min. These experimental evidences strongly implied that the annealing-induced degradation followed the manner of the latter model described above. For the photoconductivity after annealing, however, it is still uncertain whether the ratio will approach a certain constant caused by the metallic degraded layer, or converge to zero as the amorphous layer diminishes.

For a-As_2_Se_3_, no changes could be observed for the resistivity, the optical microscope observation and the transmittance. This indicated that the As content maintained its form and kept the film from reacting with substances in the atmosphere.

It is inadequate for an imaging device to have a large dark current because it is known to lose image quality and cause after-images. Although, despite the fact that a-As_2_Se_3_ has less degradation and the level of dark current is relatively stable compared to a-Se, it is still a great disadvantage for photodetector application under such small photoconductivity. In order to realize a practical photoelectric conversion device, the amount of arsenic content should be controlled and optimized to avoid degradation and maintain high photoconductivity at the same time.

## 3. Characterization of a-Se Based Photodetectors

### 3.1. Device Structure and Response Time [37,38]

**Figure 16.**
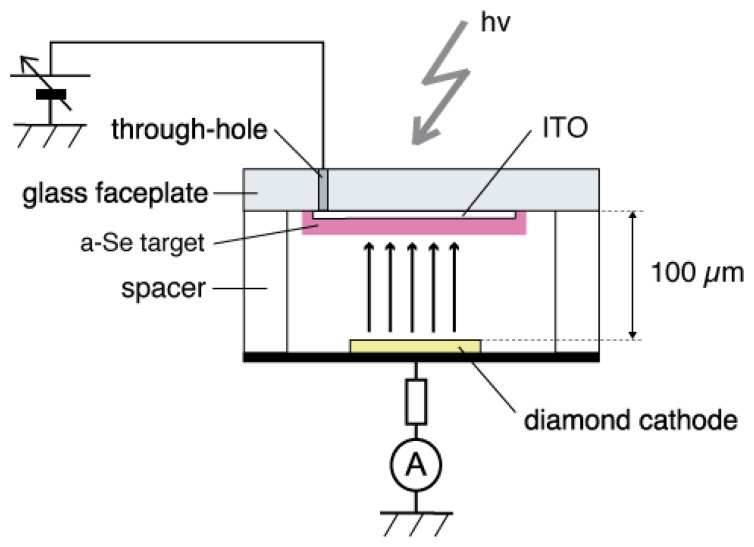
Schematic diagram of a diode-structured photodetector that consists of a-Se photoconductive anode and N-doped diamond cold cathode, after ref [37].

The a-Se anode and the diamond cathode are combined into a prototype photodetector, whose structure is illustrated in Figure 16 [37]. The anode-cathode distance was kept at 100 μm using a Teflon spacer. The setup was introduced in a vacuum chamber with a base pressure of 10^−5^ Torr, and electron emission was induced by applying voltage between the anode and the cathode. A HP 4140B was used as a voltage source, whose output was intensified 1,000-fold using a Glassman high voltage DC power supply. The emission current was measured by an Advantest R6441C digital multi-meter with a detection limit of 0.1 nA. A white light-emitting diode (LED) (Rodan Ltd., Taichung City, Taiwan, No. 10279) was fixed above the setup, and illuminated the faceplate side of the photodetector through a quarts view port. The illumination intensity at a-Se surface was estimated to be 3,600 lx when the LED was turned on, and 0.05 lx when the LED was turned off.

The operational mechanism of the photodetector can be explained as follows: electron–hole pairs are generated in a-Se film by incident illumination through the glass faceplate. Those generated holes are accelerated toward the cathode side of a-Se film by the electric field between the anode and cathode. Surface potential of a-Se rises due to the presence of accumulated holes. Consequently, the extraction field/voltage of the diamond cathode increases, and more electrons are emitted from the cathode. When the illumination is turned off, the number of holes at a-Se surface decreases because less electron–hole pairs are generated in the a-Se film. The extraction voltage of cathode, then, becomes lower and the emission current decreases. Thus, the illumination is detected as the modulation of the emission current from diamond cathode. Although this model can explain well the I-V characteristics of the photodetector, it is difficult to confirm it experimentally, since the surface potential of a-Se surface cannot be measured due to the high resistivity of a-Se film.

**Figure 17.**
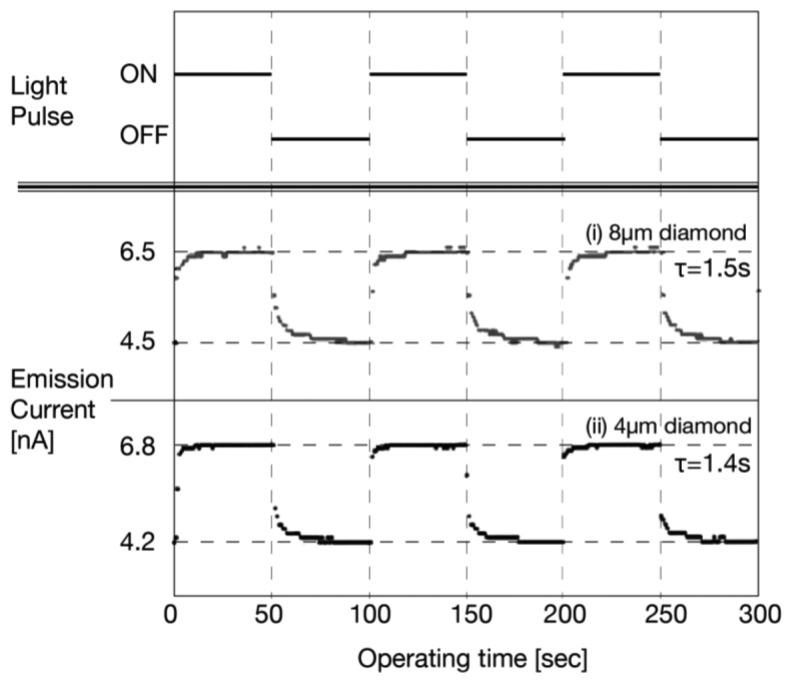
I-T characteristics of the diode-structured photodetector, which proved photo response. After Onuki *et al.* in ref [37].

The emission current (I)–operating time (T) characteristics of thick diamond (∼8 μm) and thin diamond (∼4 μm) are shown in Figure 17. The illumination was given in the pulse with an interval of 50 s, and the anode voltage was fixed to 500 V.

The photodetector exhibited stable operation for more than 3 h even in a low vacuum around 10^−5^ Torr. The emission current increased when illumination was on and decreased when illumination was off. The response time, however, was relatively slow [37].

Two possible factors were considered as the cause of this slow response. One is due to a number of trap levels in the photoconductive films. Some of the carriers induced by incident illumination are first captured in the trap levels of the photoconductor, and excited again to the conduction band. This indirect transition leads to slow response. The other is due to high resistance and capacitance in the electric circuits of imaging devices, which gives a large time constant. Time constant is a product of resistance and capacitance as in typical RC circuit. Other possibilities such as the delay of the transient illumination or the cathode response were examined and confirmed to be negligibly small compared to the observed delay in photo response.

As for the effect of trap levels in a-Se, it seemed negligible because the photo response of a-Se for a thickness of 4 μm was reported to be less than 20 ms [39]. On the contrary, the delay caused by the time constant should be examined carefully, since the resistivity of diamond was as high as 100 GΩ cm. The high resistivity was likely to result in a large time constant (*i.e.*, RC product) for the same capacitance. In order to evaluate the effect of the time constant, I-T characteristics were compared between diamond cathodes with different film thicknesses. Since the resistivity of the diamond was extremely high, the total resistance of the circuit should be reduced greatly when the diamond thickness was reduced to half. The total capacitance of the circuit, on the other hand, should show no significant difference between thick and thin cathodes.

As shown in Figure 17, the I-T characteristics using thin diamond showed almost no change compared to the that using thick diamond. The time constant was estimated to be 1.4 s for thin diamond, and 1.5 s for thick diamond. This result clearly indicated that the slow response was independent of diamond thickness. Therefore, it was likely that the high resistance and capacitance of the diamond cathode had a small effect on the response of the detector.

The results suggested that the slow response of the photodetector originated neither in trap levels in a-Se nor in large RC components. These results led to the interpretation that the slow response was due to transient surface potential of a-Se. As mentioned in the operational mechanism, the extraction field/voltage of diamond in this photodetector depended on surface potential at a-Se. The previous result for cathode response indicated that the diamond cathode should show a rapid response to the change of potential at a-Se surface. Therefore, the response of the emission current in Figure 17 represented the potential variation at the a-Se surface, in other words, the surface potential of a-Se should follow an exponential variation.

The reason for the slow transition of surface potential at a-Se was explained as follows: when illumination was on, the number of holes that accumulated at a-Se surface was increased due to the photo-generated holes, and the electrical potential at a-Se surface increased. Subsequently, the rise in surface potential enhanced the electron emission from diamond. Emitted electrons, then, compensated holes at the surface, decreasing its potential. This transient increase of potential at the surface caused the emission current to increase slowly. When compensation was balanced, the emission current reached an equilibrium state. No or less photo-generation occurred in the a-Se bulk when illumination was turned off. Accordingly, the potential at the a-Se surface decreased due to decrease in the number of holes at the surface, thus, the emission current decreased until it reached equilibrium.

**Figure 18.**
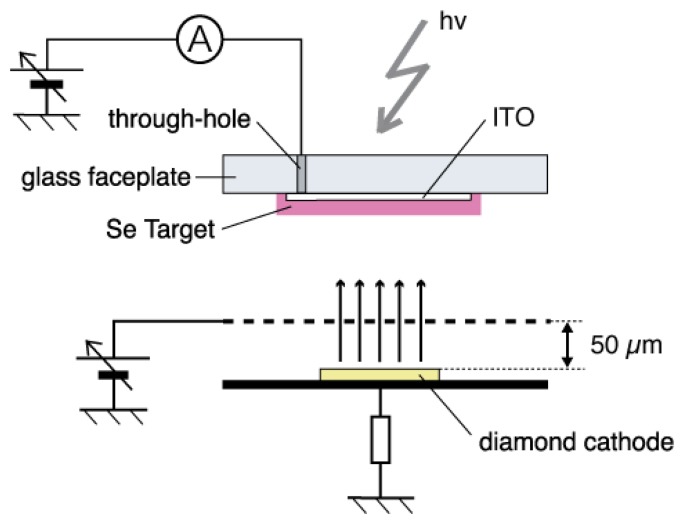
Schematic diagram of a triode-structured photodetector after Suzuki *et al* [38]. An extraction grid is introduced to control the emission current independent of a-Se surface potential.

In order to improve the response time of the photodetector, triode-structured device was proposed by Suzuki *et al* [38]. By adding extraction grid above the cathode, as illustrated in Figure 18, this device enabled one to control electron supply from the cathode without transient change of surface potential of a-Se film.

The operational mechanism of the triode-structured photodetector is as follows. The back contact of the diamond film is electrically grounded and the extraction voltage is applied to the mesh grid to extract electrons from the diamond cathode. The emitted electrons are accelerated towards the grid and some of them can reach a-Se surface through the grid. At the same time, a positive voltage is applied to the anode. Photo-generation produces electron-hole pairs in the a-Se film when illuminated by light, and the holes are accelerated downwards to the surface of the a-Se. The accumulated holes at the a-Se surface give rise to the number of electrons, which can reach the a-Se surface. The increase in electrons by incident illumination is detected as the increase in the anode current. The anode current depends not only on the illumination of the a-Se but also the anode voltage. When a higher anode voltage is applied, more holes are accelerated and accumulated at the a-Se surface. The anode voltage can continuously control the dynamic range of the anode current to the illumination.

**Figure 19.**
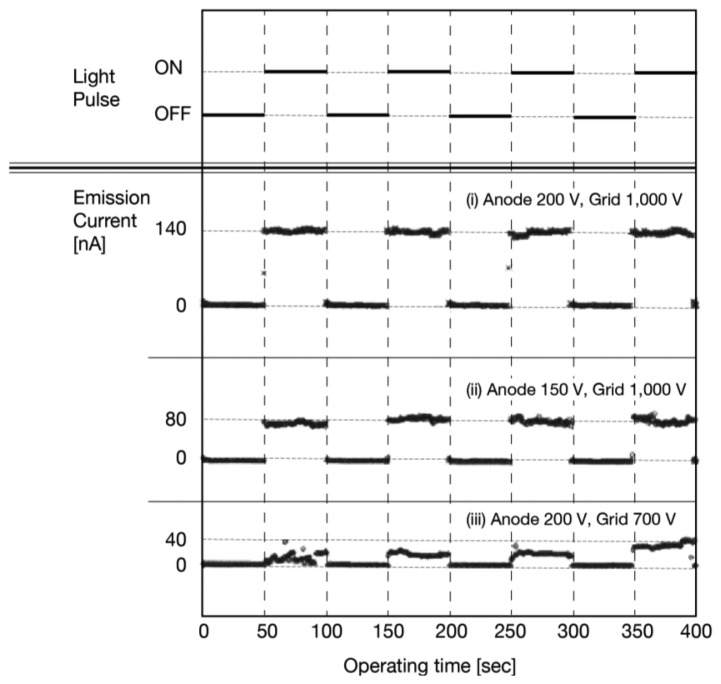
I-T characteristics of triode-structured photodetector after Suzuki *et al.* [38]. The bias conditions were (**a**) anode voltage 200 V and grid voltage 1,000 V, (**ii**) anode voltage 150 V and grid voltage 1,000 V, (**iii**) anode voltage 200 V and grid voltage 700 V.

Figure 19i shows the photo-response of the anode current. The anode voltage was fixed to 200 V and the grid voltage was 1,000 V. The illumination was given in pulse with intervals of 50 s. This result clearly indicated successful operation of the photodetector. The anode current rapidly increased when the illumination was ON and decreased when OFF. Simultaneous measurement on the cathode current confirmed that the cathode current did not show photo-response by the incident illumination, which supported the suggested operation mechanism.

Figures 19ii,iii show the result of the same measurement with different anode voltages. The anode voltage was reduced to 150 V with the same grid voltage as Figure 19ii. The anode current, when the illumination was ON, was reduced compared to Figure 19i. This result indicated a variable dynamic range of this photodetector. The number of holes at the a-Se surface should be decreased by lower anode voltage as long as the grid voltage was fixed. A future a-Se imaging device would be able to observe an image in a wide range of ambient illumination intensity by controlling the anode voltage, when this photodetector was made into a unit pixel of the a-Se imaging device.

The grid voltage was, then, set at a reduced level to establish the effect of the electron beam current. Figure 19iii shows the photoresponse of the anode current with a lower grid voltage. The anode voltage was 200 V, which was the same as in Figure 19i, but the grid voltage was reduced to 700 V. The dynamic range of the anode current to the illumination intensity had greatly decreased compared to Figure 19i. Moreover, the fluctuation on the anode current was larger than that of Figure 19i with the incident illumination. These results indicated that a stable photo-response of the detector required sufficient amounts of electrons from the diamond cathode towards the a-Se film. Conceivably, a lower grid voltage could give proper operation of the photodetector as long as sufficient amounts of electrons were reaching the a-Se surface by using a lower threshold-voltage diamond cathode.

The diode-structure photodetector, without mesh grid, is a possible candidate to avoid the loss of electron beam current by the grid [37]. In a diode-structure photodetector, the operational mechanism is completely different from that of triode-structure. The extraction voltage of a diamond cathode cannot be controlled independently of the acceleration voltage across the a-Se film in diode-structure. This independence is not suitable for the fast photo-response to incident illumination, and the variable dynamic range of the detector. The triode-structure is, therefore, superior to the diode-structure in terms of both fast photo-response and variable dynamic range to incident illumination.

The triode-structure photodetector successfully responded to the incident illumination with variable dynamic range. However, the electrons captured by the mesh grid caused the shortage of electron beam current for stable photodetector operation with a lower grid voltage. Further careful investigation of the grid mesh structure would be necessary to lower the operational voltage of the photodetector, along with maintaining sufficient electron beam current from the diamond cathode.

### 3.2. Sensitivity to the Light with Different Wavelength Range [40,41]

**Figure 20.**
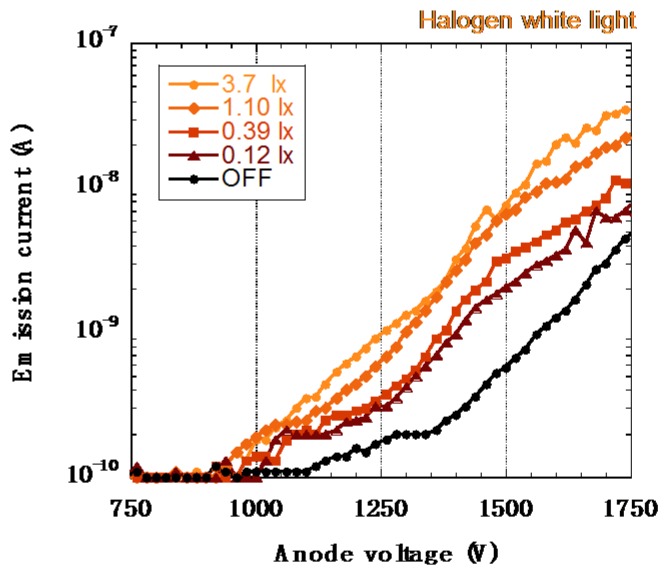
I-V characteristics of a-Se based photodetector under different lighting conditions [40].

Sensitivity of the a-Se based photodetector was evaluated by Kato *et al.*, using emission current (I) *versus* anode voltage (V) characteristics [40]. Figure 20 shows typical I-V characteristics of the detector when illuminated by non-filtered halogen light. Each line represents a characteristic I-V curve under a fixed intensity of light. It clearly showed an increase of the emission current when the light was turned on. The authors could hardly observe any aging phenomenon of this detector throughout numerous measurement attempts. The aging phenomenon here meant that the film no longer exhibited either emission current or photoconductivity. After it was continuously used for tens of hours, however, the signal current when the light was on did seem to gradually decrease. Nonetheless, this result showed that the incident light induced the increase in the emission current: this change in the emission current by light illumination was defined as the photo-response. The photoresponse occurred for light intensities as low as below 0.39 lx. This low illuminance of 0.39 lx was approximately the same to the brightness under moonlight. It was usually difficult to obtain clear image under this low illumination using a normal silicon based devices such as CCDs or CMOS sensors unless it was cooled down by a Peltier device or by liquid nitrogen in order to prevent any noise interfering the image.

**Figure 21.**
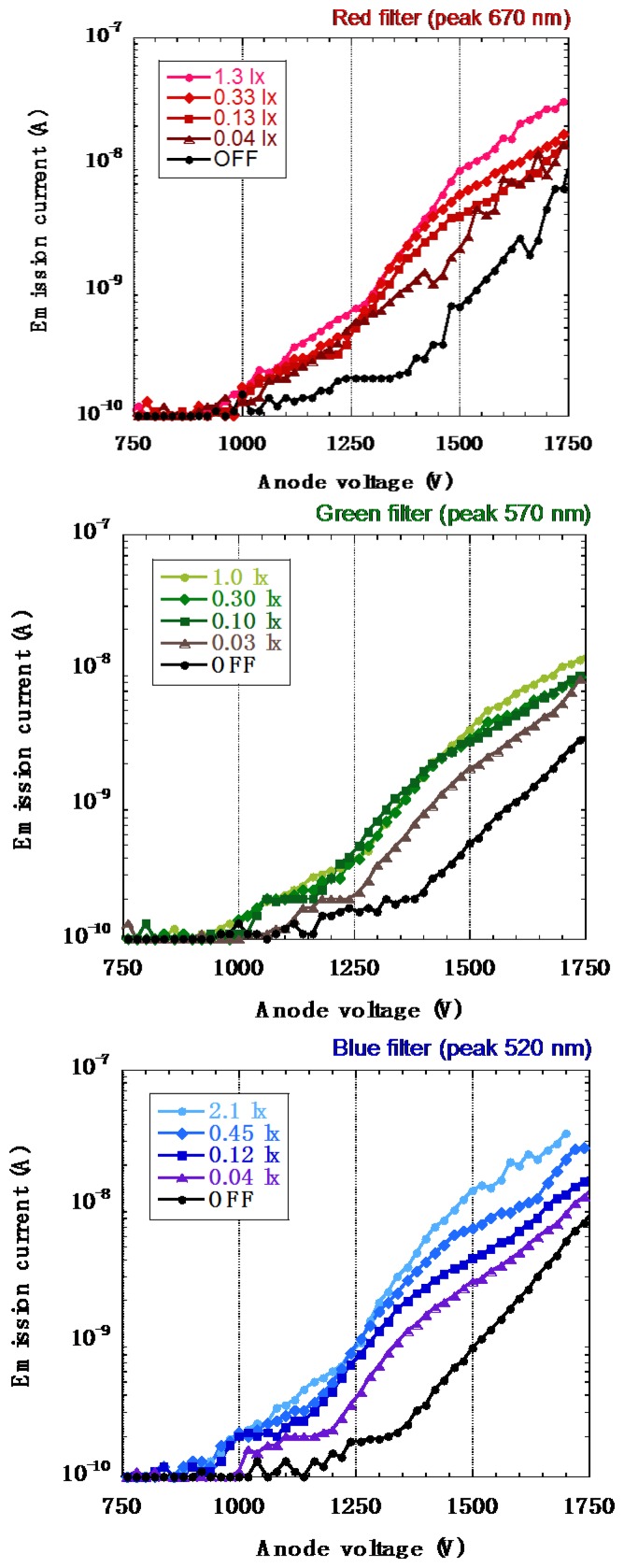
I-V characteristics of a-Se based photodetector illuminated using different color filters: (**a**) red with peak wavelength 670 nm, (**b**) green with peak wavelength 570 nm and (**c**) blue with peak wavelength 520 nm [40].

The same I–V measurements were then conducted for different wavelengths: red, green and blue filtered light were used to illuminate the detector, each under the same manner. Measuring photo-response towards three primary colors of light is obviously essential for future applications and practical usage. Figures 21a–c are the I–V characteristics under red (R), green (G) and blue (B) lights, respectively. The peaks for red, green, and blue lights were at 1.85 eV (670 nm), 2.17 eV (570 nm), and 2.38 eV (520 nm) respectively. Although a-Se is reported to have an optical band gap of 2.0 eV and cannot detect red light [4], this a-Se film is modified by tellurium doping. Doping tellurium (Te) is known to narrow the band gap of a-Se down to 1.8 eV, which enables photoconductive film to detect red light.

The emission current corresponding to the red light was almost equivalent to the emission current measured under green or blue light for the same illuminance. This result showed that Te intensified the photo-response towards red light when it was successfully doped into a-Se, where a-Se without doping Te could not detect red light illumination.

**Figure 22.**
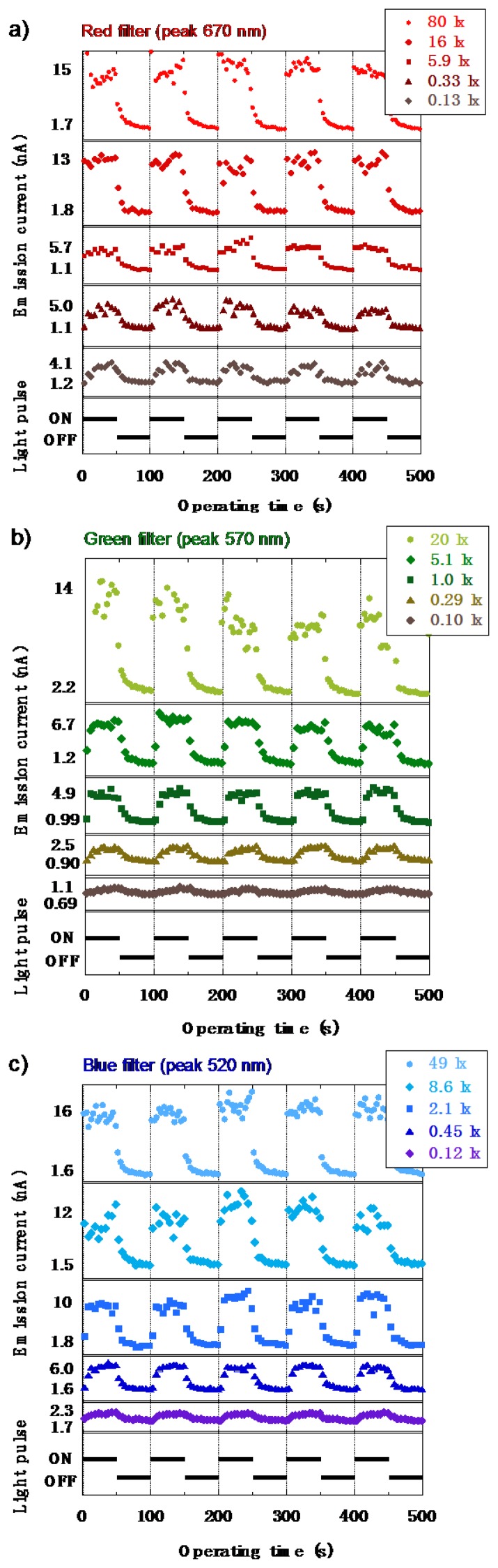
I-T characteristics of a-Se based photodetector illuminated with filtered halogen lamp, reported in ref [40]. Peak wavelengths of the lights were (**a**) 670 nm for red filter; (**b**) 570 nm for green and (**c**) 520 nm for blue.

The characteristics of the emission current (I) *versus* the operating time (T) under RGB light are shown in Figures 22a–c, respectively. The anode voltage was fixed at 1,500 V, and the RGB illumination was given in 10 mHz pulse. The results showed a similar result observed in the I–V characteristics where an increase of the emission current was observed even for the low illumination of 0.1 lx. When the illuminance was higher, the difference between the dark current and the light current became larger. However, the photoresponse was slightly slow, which was caused by the transient surface potential of a-Se based film due to diode structure [37]. Triode structure, which could control the extraction voltage independently from the anode voltage, was expected to improve slow response [38]. In a triode- structure photodetector, a mesh grid was inserted between the anode and the cathode. The voltage was applied to the grid independently from anode voltage, which extracted electrons from diamond cathode and controlled the emission current. This time the signal current was measured in the anode side. Triode structure, thus, could enable faster response than diode structure.

**Figure 23.**
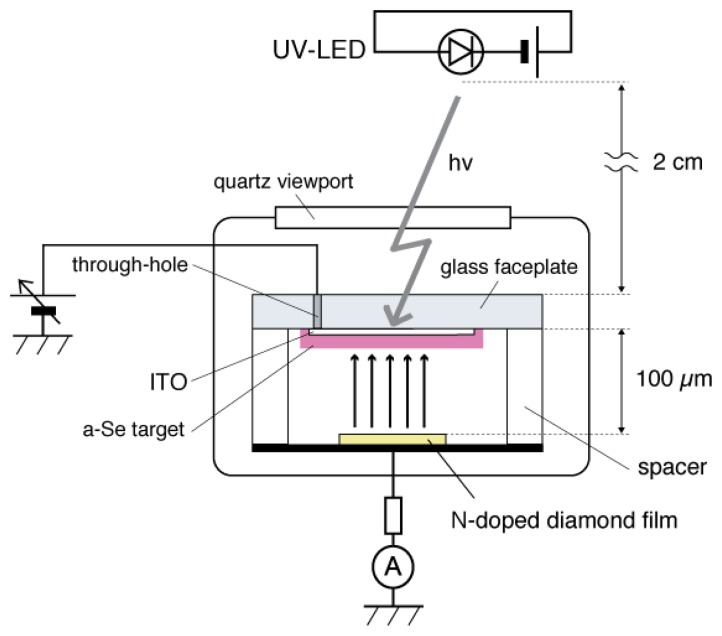
Schematic diagram of diode-structure photodetector that consist of a-Se photoconductor and N-doped diamond cold cathode [41].

**Figure 24.**
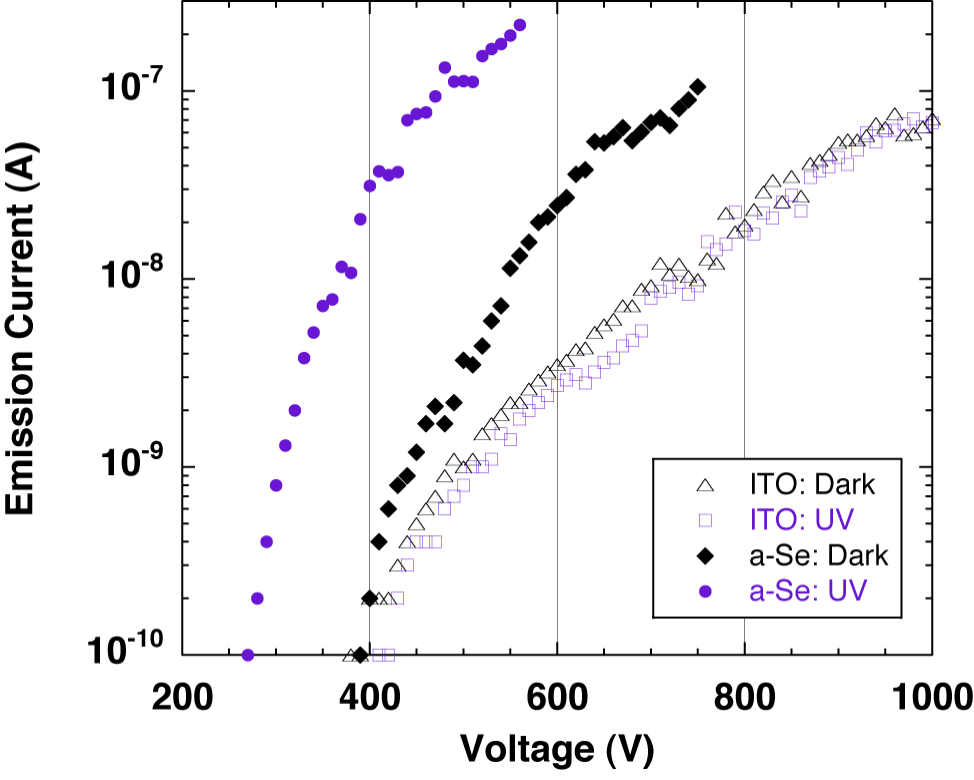
I-V characteristics of a-Se based photodetector under different anode and UV illumination conditions, after ref [41].

The sensitivity to the UV light was confirmed by the emission current−applied voltage (I–V) characteristics of the detector. Figure 23 shows the schematic setup for the measurement system. The I–V characteristics were compared between with UV illumination and without illumination, which were illustrated as a-Se:UV and a-Se:Dark in Figure 24. The I–V curves showed that the emission current at a fixed voltage (e.g., ∼400 V) increased by up to two orders of magnitude under UV illumination, compared to the dark current [41]. This proved a successful UV detection. The response to the UV light was also confirmed by periodic UV illumination while monitoring the emission current at fixed voltage. In the discussion below, the applied voltage, where emission current of 0.1 nA was observed, was defined as the threshold voltage for this device. When the light source was turned off, the threshold voltage was 400 V, and the emission current increased exponentially as applied voltage increased. However, the current began to saturate after 10 nA and eventually became linear. On the contrary, when the setup was illuminated by UV light, the threshold voltage was reduced to 300 V, and linear component was much smaller than the characteristics without UV illumination.

Although the changes in I-V characteristics with/without UV illumination was mostly due to the carrier excitation in a-Se film, there were possibilities that these changes were caused by photoemission from the diamond surface. In order to measure this photoemission current from the cathode, I-V measurements with/without UV illumination were conducted using the same cold cathode but without the a-Se using ITO as a transparent anode. The geometrical configuration of the detector was kept the same as before. The obtained I-V curves, illustrated as ITO:UV and ITO:Dark in Figure 24, showed no differences regardless of UV illumination, which negated the possibility of the photoemission from the cathode.

It should be noted the slopes of the I-V characteristics at current above 10 nA were similar regardless of the anode or UV illumination, which suggested that the emission current was limited by a common factor-possibly by a series resistance. The series resistance of the measurement system calculated from the I-V curves were in the range of 0.9–4.2 × 10^9^ Ω for all measurement conditions. This current saturation was speculated as current limitation by bulk resistance of the diamond [42–44], which was often observed in the I-V characteristics of diamond cold cathodes with bulk resistance of over 10^9^ Ω.

The different I-V characteristics at relatively low voltage region were explained by carrier multiplication in a-Se. When ITO was used as the anode, carrier multiplication did not occur in the anode. The potential difference between ITO surface and the cathode was almost same as the applied voltage, therefore, the I-V characteristics followed the same curve regardless of UV illumination. The threshold voltage shown in the I-V curves were identical to the extraction voltage of the diamond film.

On the contrary, when a-Se was used as the anode, the potential of a-Se surface depended on the UV illumination, and was assumed to be slightly higher than the applied voltage. The reason to this was as follows: when the detector was illuminated by UV light, electron hole pairs were generated in a-Se film and under the application of a sufficient voltage (∼300 V in this study) between the anode and the cathode, some of the generated electron hole pairs became carriers, of which holes induced carrier multiplication while drifting to the cathode side of the a-Se film. As a result, carrier multiplication of holes took place, and those accumulated in the cathode side of the a-Se film enhanced surface potential of a-Se. The increased surface potential led to electron emission at voltage lower than the usual extraction voltage. In contrast, when the detector was not illuminated, photo-induced electron hole pairs were not expected in a-Se film, and the surface potential at a-Se film was not enhanced. As the applied voltage grew large and exceeded the extraction voltage, electrons were emitted from the cathode and injected into a-Se. The energy of the injected electrons was large enough to induce carriers in a-Se, which led to multiplication. The dark current of the detector was therefore higher than the dark current measured with ITO anode.

Based on the discussions above, it was found necessary to prevent carrier injection into a-Se film in order to maximize the signal gain of a-Se based photodetector. Possible solutions were to deposit a blocking layer on top of a-Se photoconductor [1], as well as use of low-energy electrons for signal read-out. Since heavily N-doped diamond was reported to operate with battery power, it was a possible candidate for this application. Use of retarding electrode was another possible solution to avoid carrier injection.

## 4. High-Sensitivity Photo Detection Using Carrier Multiplication [45]

Although the unique carrier multiplication should lead to ultra high-sensitivity photo imagers, the unknown physics of carrier multiplication limited application of this technology in visible light; so far, a lucky drift carrier multiplication mechanism was proposed through empirical calculations and simulations. In this chapter, we investigated an alternative model of carrier multiplication phenomenon in a-Se film. Our research work produced an a-Se device having four times the signal gain as compared with a standard HARP. In addition, Raman spectroscopy and time-of-flight (TOF) secondary ion mass spectroscopy (SIMS) measurements were utilized to understand the conditions needed to induce the multiplication.

The depth profile of the film composition was characterized using time-of-flight secondary ion mass spectroscopy (TOF-SIMS) [45]. In the TOF-SIMS measurement, the surface of the film was alternately bombarded by ion beams for etching and analysis, which enabled one to take a depth profile of the composition. The measurement was conducted using ION-TOF, TOF.SIMS5 system, under the following conditions: Ga, and Cs ion beams were used to analyze and sputter etch the sample, respectively. The Ga ion gun was operated at an acceleration voltage of 25 kV, and the beam current was 1.5 pA. The Cs sputter beam was operated at acceleration voltage of 1 kV and beam current of 4 nA. The spot size of the analysis beam was set to 100 μm × 100 μm and the sputtered area was 200 μm × 200 μm. Both analysis beam and sputter etching beam were alternately irradiated for 4 s. Since this measurement was destructive, the analysis area was marked and avoided for further analysis.

**Figure 25.**
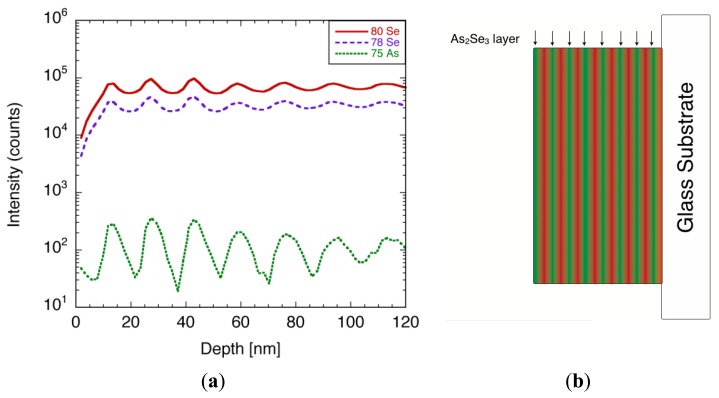
(**a**) Typical TOF-SIMS spectrum of As-incorporated a-Se film. (**b**) Schematic diagram explaining the structure of the deposited film using Se and As_2_Se_3_ as evaporation source [45].

A typical TOF-SIMS spectra of the film, focusing on ^79^Se, ^80^Se, and ^75^As, is illustrated in Figure 25a. Both Se and As signals showed oscillating peaks, whose number matched the number of times the film swept over each of the evaporation boats during the film deposition. The thickness of each layer was calculated from the etching rate, and was estimated to be 15 nm. Since the film consisted of Se layers and As-rich Se layers, there should be a phase shift between Se and As signals. However, the shift was less visible due to the angle of the sputtering beam allowing the alternative layer to etch away on its way, which led to the modulation in the signal. From the TOF-SIMS spectra, it was evident that the films had multi-layered structure with different arsenic content, as shown in Figure 25b. A quantitative analysis of arsenic concentration was difficult at present, due to the lack of reference samples.

In order to confirm the effect of As incorporation, the structures of the films were characterized using Raman spectroscopy. It was reported in the previous studies that a-Se recrystallized into t-Se by laser illumination in Raman spectroscopy system [34]. It was also known that the recrystallization was induced by thermal treatment such as annealing at temperature higher than 65 °C, whose details were analyzed elsewhere [15].

The a-Se films with different As content were measured by Raman spectroscopy, and changes in the spectra were compared for different signal accumulations. The measurement system used in this study was triple monochrometer Raman system (Dilor XY) equipped with a CCD camera for multi-channel detection. The deposited films were placed in an ultra high vacuum (UHV) chamber, and were illuminated by an excitation laser, which was the 1.92 eV (647.1 nm) emission line of a Kr ion laser. The monochrometer slits were set for a resolution of 2.4 cm^−1^. The laser with a power of 18 mW was focused to a spot of approximately 200 μm in diameter on the sample surface. All Raman spectra were recorded in a backscattering geometry. A detailed description of this set-up was given in [34].

**Figure 26.**
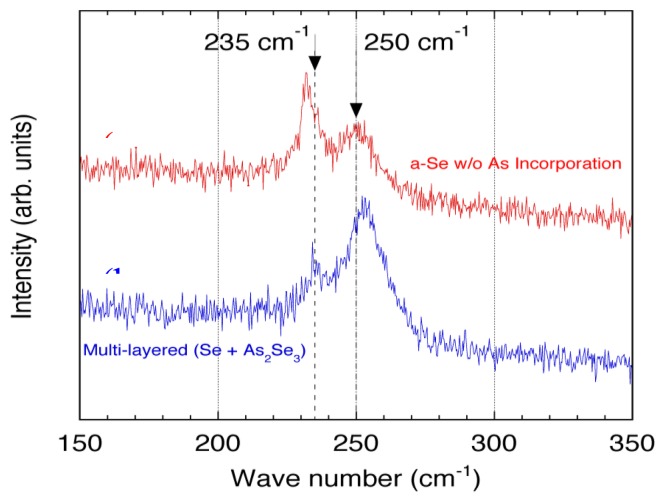
Raman spectra of the deposited film: (**a**) without As incorporation and (**b**) with As incorporation [45].

Figure 26 illustrates the Raman spectra for the film with and without As incorporation [45]. The relatively broad feature with a peak at 250 cm^−1^ was characteristic to a-Se, and the sharp peak at 235 cm^−1^ represented t-Se, which was produced by crystallization. The results shown in Figure 26 demonstrated that the a-Se peak in the As incorporated film remained even after 51 accumulations. In contrast, Raman spectrum for the pure a-Se film showed that the a-Se peak decreased rapidly after 15 accumulations and almost disappeared after 39 accumulations. The crystalline t-Se peak at 235 cm^−1^ appeared and dominated the spectrum after 51 accumulations, meaning that the film had almost entirely crystallized into t-Se.

**Figure 27.**
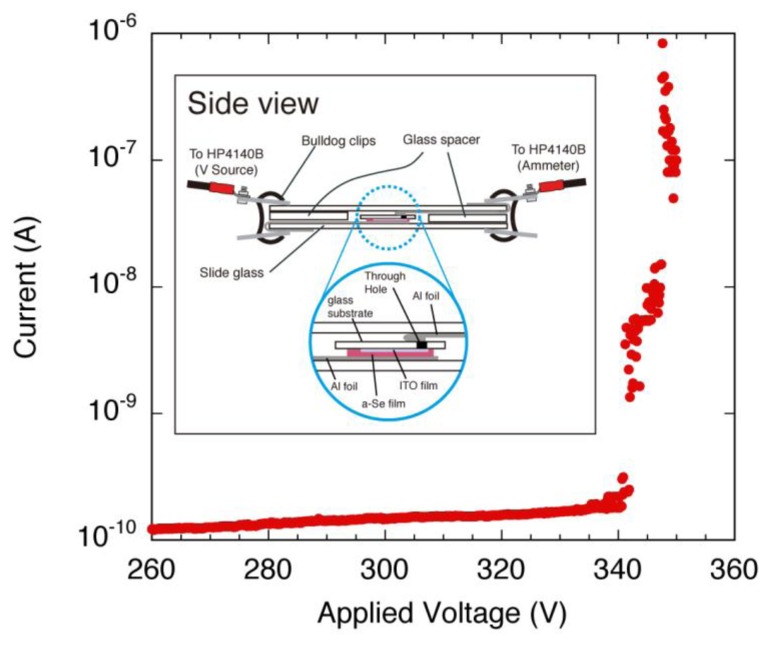
A typical contact current-applied voltage (contact I-V) characteristics of a-Se film with As incorporation [45]. The experimental setup is depicted in the inset.

In order to confirm the carrier multiplication phenomenon, the electronic properties of the multi-layered films were evaluated by contact current (I)-applied voltage (V) measurements. The measurement system used for contact I-V measurement was depicted in the inset of Figure 27. This system was specially designed to characterize high resistive films, with average noise levels as low as 10^−14^ A. A white halogen lamp was used to illuminate the samples. The illuminance of the lamp was approximately 2,000 lx. A typical I-V characteristic of the film is shown in the same figure. The characteristic I-V curves showed a linear current increase as the applied voltage was increased from 0 V. A drastic current increase was observed at 340 V, at which point I-V curve became exponential. This transition in current increment was observed in each of the iterative measurement on the same sample, thus the transition was not due to the irreversible breakdown. The resistivity of the film was estimated to be as high as 1.6 × 10^16^ Ω cm, using I-V curve at voltage lower than 340 V and assuming contact area to be 2 cm^2^. The extremely high resistivity of the film was attributed to low carrier concentration due to high trap density.

In contrast to the As-incorporated films, the contact current was larger by up to two orders of magnitude for pure a-Se films, and high voltage could not be applied due to the current limit. These films were possibly crystallized in the first few measurements, and ended up having a resistivity lower by orders of magnitude than that of amorphous films. Such a low-resistive film was not suitable for high-sensitivity photo detection: the carrier multiplication took place under strong electric field of more than 80 V/μm, and a high-resistivity was essential to maintain this strong electric field. The Raman spectra clearly showed that the As incorporation was effective to maintain high resistive amorphous structure that was suitable for the carrier multiplication.

**Figure 28.**
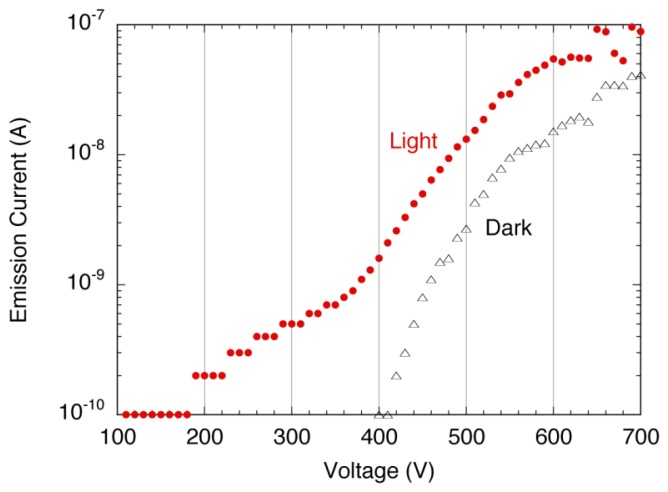
A typical emission current-applied voltage (I-V) characteristics of a-Se film with As incorporation [45].

The As-incorporated a-Se film was then combined into a prototype photodetector, along with N-doped diamond serving as a cold cathode. Figure 28 shows the typical emission current-applied voltage characteristic of the photodetector. A signal to noise (S/N) ratio at voltage below 400 V was very high, due to low dark current of as low as detection limit (0.1 nA). When the white light was turned on, the emission current is observed from 100 V, which was well below the threshold voltage of the emitter.

The reason of the low threshold voltage under light illumination might be that carriers were excited by the incident light and positive holes accumulated on the film surface. The accumulated holes increased the potential of the film surface, which induced the electron emission from the emitter at voltage lower than its extraction voltage. The emission current showed a steep rise at around 340 V under light illumination; however, the current increase was limited compared to the contact I-V characteristic (Figure 26). As the voltage increases, saturation of the current was more obvious, and S/N ratio decreased. The cause of the current saturation could be limited current supply from the emitter. Either high bulk resistance or carrier depletion in the emitter was suspected. A solution of this current limitation was introducing heavily nitrogen (N)-doped diamond as electron emitter. It was reported that the emission current from heavily N-doped diamond was limited by electron injection between back contact and diamond film [32], which should prevent carrier depletion. The solutions to the current saturation, as well as improvement in S/N ratio at high current are under investigation.

The sensitivity of the photodetector was estimated from the emission current-applied voltage characteristics. An increase in current density Δ*i* (Acm^−2^) in a photoconductor was usually expressed as follows:

(1) $$\Delta i=e\mu \tau \eta N_{0}(1-\exp(-\alpha D))E$$

where *E* (Vcm^−1^) is the electric field across the photoconductor, *e* (C) is the elemental charge, *μ* (cm^2^sec^−1^V^−1^) and *τ*(sec) are the mobility and lifetime of the carriers, *η*(unitless) is the quantum efficiency, *N*_0_ (sec^−1^cm^−3^) is the number of incident photons captured in the photoconductor per unit time. The term exp(−*αD*) represents the fraction of photons that penetrate the film: this term depends on the product of *α*(μm^−1^) and *D* (μm), which are the absorption coefficient and the thickness of the photoconductor. Reflection was assumed to be negligibly small. The product *μτE*, or carrier Schubweg, was known to be a limitation factor when carrier path was long compared to the Schubweg. In the present case, however, the thickness of the film was 2 μm, which was small enough to ignore the limitation from Schubwegs. Previous study reported that the Schubweg of a-Se was 1.2 mm–12 mm for holes and 0.3 mm–1.2 mm for electrons [13]. The contribution of *μτE* to the current was, therefore, assumed to be negligible.

Based on the assumptions above, nominal quantum efficiency, *η_nom_* (unitless), was introduced to evaluate the sensitivity of the detector from measurable parameters. The definition of the nominal quantum efficiency was as follows:

(2) $$\eta_{\text{nom}}=\frac{\Delta I}{eN_{0}\;(1-\exp(-\alpha D))}$$

where current increase is represented by Δ*I* (A) and other parameters taken from Equation (1). Here we assume that all the incident electrons are absorbed within the photoconductor, so that the term exp(−*αD*) becomes zero: this assumption gives smallest nominal quantum efficiency. Using this assumption, the nominal quantum efficiency can be estimated from current and the number of incident photons.

In the emission current-applied voltage characteristic of the photodetector, 1 nA of current increase was observed at 400 V. The number of incident photons was roughly calculated assuming the wavelength of incident light to be 555 nm. The assumption was valid considering the mobility gap of a-Se, which was 2.0–2.2 eV without incorporation of tellurium. Providing this condition, illuminance of the incident light was equivalent to 55.48 J/m^2^s, which were 1.55 × 10^16^ photons per square centimeters per second. The area of the detection spot was estimated from the spot size of the emitter. It was reported that the emission area was smaller than 0.1% of the emitter area, and was a few micrometer square in this study. Substituting these parameters to the Equation (2), the nominal quantum efficiency was calculated to be 10–40. This meant that 10 to 40 carriers were created per incident photon, which was possible only if signal multiplication took place. In the previous research it was reported that the HARP mode photodetector showed quantum efficiency of 10 [1], which was in good agreement in our results.

## 5. Conclusions

As a conclusion, a-Se based photodetectors were reviewed, and development of a-Se photodetector was attempted using N-doped diamond cold cathode as signal readout. High sensitivity photo detection was demonstrated using As-incorporated a-Se film as photoconductor, which resulted in quantum efficiency of 10–40 for visible and 1,000 for UV light.

## Figures and Tables

**Table 1.** Electron hole properties of undoped and stabilized a-Se, after ref [13].

**Property**	**Units**	**Undoped a-Se**	**Stabilized a-Se**
Band gap (optical)	eV	2.0	2.0
Band gap (mobility)	eV	2.1–2.2	2.1–2.2
Hole mobility	cm^2^V^−1^sec^−1^	0.13–0.14	0.13–0.14
Hole lifetime	μsec	10–100	50–500
Electron mobility	cm^2^V^−1^sec^−1^	5–7 × 10^−3^	2–4 × 10^−3^
Electron lifetime	μsec	10–100	200–1,000
